# Machine learning identifies the association between second primary malignancies and postoperative radiotherapy in young-onset breast cancer patients

**DOI:** 10.1371/journal.pone.0316722

**Published:** 2025-02-06

**Authors:** Yulin Lai, Peiyuan Huang

**Affiliations:** 1 Department of Urology, The People’s Hospital of Longhua, Shenzhen, P. R. China; 2 Department of Pharmacy, Gaozhou People’s Hospital, Maoming, P. R. China; King’s College Hospital NHS Trust: King’s College Hospital NHS Foundation Trust, UNITED KINGDOM OF GREAT BRITAIN AND NORTHERN IRELAND

## Abstract

**Background:**

A second primary malignant tumor is one of the most important factors affecting the long-term survival of young women with breast cancer (YWBC). As one of the main treatments for breast cancer YWBC patients, postoperative radiotherapy (PORT) may increase the risk of second primary malignancy (SPM).

**Methods:**

Machine learning components, including ridge regression, XGBoost, k-nearest neighbor, light gradient boosting machine, logistic regression, support vector machine, neural network, and random forest, were used to construct a predictive model and identify the risk factors for SPMs with data from the Surveillance, Epidemiology and End Results. Multivariate logistic regression analysis was used to assess the risk of SPM associated with PORT. The cumulative incidence of SPMs was determined by competing risk regression analysis.

**Results:**

Among the 44223 YWBC patients included in our study, 3017 developed SPMs. Among all the clinical characteristics, PORT was the most common SPM. YWBC patients receiving PORT had significantly greater risks of second primary solid malignancies (SPSMs, RR = 1.61), including breast cancer (RR = 1.89), lung cancer (RR = 2.12) and thyroid cancer (RR = 1.48), but not second primary hematologic malignancies (RR = 1.32; 0.94–1.88). SPSMs were more common in YWBC individuals who were black, had a lower median household income and had fewer lymph nodes examined. Additionally, we developed a prediction nomogram with an area under the curve of 0.75 to assess the likelihood of developing SPMs.

**Conclusion:**

YWBC patients receiving PORT had a greater risk of developing SPSMs (thyroid, lung, and breast cancer), indicating the necessity of long-term surveillance of these patients. Standard adjuvant PORT should not be recommended for breast cancer patients with favorable histology and a low risk of relapse.

## Background

Breast cancer is a heterogeneous illness influenced by both genetics and the environment [1]. The incidence of breast cancer continues to rise despite decades of laboratory and clinical studies [2]. Decreased fertility and increased obesity are contributing factors to the increasing incidence of breast cancer [3]. Breast cancer accounts for 313,510 new cases and 42,780 deaths annually in America [4]. Young women with breast cancer (YWBCs) are rare, affecting only 4–6% of women under 40 years of age. The risk factors and molecular mechanisms for YWBC patients are far from clear. Some studies have shown that TP53 and PIK3CA mutations are relatively common in YWBC patients [5]. Moreover, changes, including angiogenesis, inflammation and extracellular matrix alterations present during pregnancy and breastfeeding, may contribute to the oncogenesis of breast cancer in young women [6,7]. Tumors with a relatively high incidence of unfavorable clinicopathologic characteristics are relatively common in YWBC patients [8]. Thus, Unfavorable prognoses are obtained in YWBC patients compared with older women. Among all therapeutic modalities, radiotherapy is thought to be appropriate for all stages of YWBC disease [9]. However, when tissues outside of treatment fields are exposed to more radiation, radiotherapy may increase the risk of second primary malignancy (SPM), especially in young cancer patients, whose tissues are susceptible [10,11]. These findings highlight the need to investigate the relationship between the PORT of YWBC and SPM risk, as this could yield further guidelines for better results and follow-up strategies.

One of the main reasons that cancer patients die over an extended period of time is SPM [12]. SPM is common in female patients with breast cancer and is associated with a poor prognosis [13]. A previous study demonstrated that receiving radiation therapy for breast cancer does not increase the chance of developing lung cancer [14]. However, another study revealed that second primary lung cancer became more common over time in breast cancer patients who had received radiation treatment [15]. Moreover, breast cancer patients receiving adjuvant radiotherapy have increased risks of contralateral breast cancer, lung cancer, and urinary system cancer [16]. Bazire et al reported that receiving radiotherapy in breast cancer patients was correlated with a higher risk of sarcoma but not with breast cancer or lung cancer [17]. This conflicting evidence may be due to the long follow-up time and the large number of cases required to study SPM. To the best of our knowledge, no study has fully clarified the correlation between PORT and the risk of SPM among YWBCs in the early stage.

The current study used information from the Surveillance, Epidemiology, and End Results (SEER) database to conduct a thorough analysis of the relationship between PORT and SPM incidence among early-stage-onset YWBC patients. Our findings could offer additional guidance for successful interventions aimed at preventing SPM as well as treatment and follow-up plans for YWBC patients.

## Methods

### Patient acquisition and screening

The initial main YWBC cases diagnosed between 1975 and 2020 were gathered from the SEER database. The combined SEER summary stage and historic stage A variables were used to determine the clinical stage of YWBC patients. The inclusion criteria for YWBC patients were as follows: (1) first primary breast cancer; (2) age ranging from 18–40 years; (3) complete and reliable follow-up data; (4) YWBC patients who received PORT or did not have any radiation records; (5) localized or regional stage; and (6) female sex. The exclusion criteria were as follows: (1) had distant metastasis at diagnosis; (2) were followed up for less than 2 years; and (3) did not have complete and reliable clinical characteristics. Experts agree that a 40-year-old age restriction should be used to identify YWBC in light of the biological perspective [18]. Sensitivity analyses were carried out in our study on YWBC instances in patients who received PORT before the age of 40. Because the data were extracted from a public, deidentified database, this study did not require approval from an institutional review board.

### The exposure and interesting outcomes of our study

The exposure of our study was PORT, which was defined as receiving radiotherapy after surgery. SPMs, which include second primary solid malignancies (SPSMs) and second primary hematologic malignancies (SPHMs), were the intriguing outcomes of our study. Given the short latency time for radiation-induced carcinogenesis, two and five years, respectively, passed after the diagnosis of breast cancer before SPHMs and SPSMs were discovered and identified [19]. The diagnosis of SPM, death, or the last follow-up date, whichever occurred first, served as the follow-up endpoint.

### Machine learning component

A total of 8 machine learning components, including ridge regression, XGBoost, k-nearest neighbor, light gradient boosting machine, logistic regression, support vector machine, neural network, and random forest, were used to construct the predictive model for SPM and identify the risk factors for SPM. All these machine learning algorithms were performed according to previous studies [20–22]. The Shapley additive explanations (SHAP) method was integrated into a pipeline and embedded in machine learning components for model interpretation. The details of how the machine learning algorithm runs are shown in the S1 File.

### Statistical analysis

To internally quantify the risks of SPM based on receipt of PORT (yes vs. no), univariate and multivariate logistic regression analyses were carried out, and relative risks (RRs) and 95% confidence intervals (CIs) were calculated. Multivariate analyses were performed after adjusting for age, median household income, race, urban rural distribution, year of diagnosis, tumor grade, clinical stage, T stage, N stage, estrogen receptor (ER) status, progesterone receptor (PR) status, lymph node examination, and chemotherapy. Using the competing risk models developed by Fine-Gray, the cumulative incidence of SPM by time after the diagnosis of YWBC was computed. R software’s "rms" and "survival" packages were used to create nomograms. ROC curves and calibration curves were created to assess the performance of the nomogram. The nomogram’s clinical advantages were assessed through decision curve analysis (DCA). R 4.4.0, SPSS version 22.0 and GraphPad Prism 10.0 were used for the statistical analyses.

## Results

### Overall characteristics of YWBC patients

Table 1 shows the characteristics of the 44,223 YWBC patients included in our study. Approximately 74% (n = 32,904) of the YWBC patients were White. Most YWBC cases were estrogen receptor (68%) and progesterone receptor (61%) positive. The median follow-up time was 10 years. A total of 23,394 patients were in the localized stage, and 20,829 patients were in the regional stage. Among these YWBC patients, 22,620 (51%) received PORT. A total of 1230 and 1787 YWBC cases, accounting for 6% and 8% of all YWBC cases in the no PORT group and PORT group, respectively, developed SPMs over the follow-up period (Table 1).

**Table 1:** The clinical characteristics of the breast cancer patients included in our study.

Variables	Total, no. (%)	No PORT, no. (%)	PORT, no. (%)	P value
Total No. of events	44,223	21,603	22,620	
Median follow-up time, Years (IQR)	10 (6, 16)	10 (6, 16)	10 (5, 16)	0.464
Age at diagnosis				0.532
18–30	4,838 (11%)	2,417 (11%)	2,421 (11%)	
31–40	39,385 (89%)	19,186 (89%)	20,199 (89%)	
Medium household income				< 0.001
$75,000-	22,681 (51%)	11,584 (54%)	11,097 (49%)	
$75,000+	21,542 (49%)	10,019 (46%)	11,523 (51%)	
Race				0.093
White	32,904 (74%)	16,195 (75%)	16,709 (74%)	
Black	5,766 (13%)	2,791 (13%)	2,975 (13%)	
Others	5,553 (13%)	2,617 (12%)	2,936 (13%)	
Urban rural distribution				0.492
Metropolitan	40,415 (91%)	19,743 (91%)	20,672 (91%)	
Nonmetropolitan	3,808 (8.6%)	1,860 (9%)	1,948 (9%)	
Year of diagnosis				< 0.001
1975–1999	6,682 (15%)	3,484 (16%)	3,198 (14%)	
2000–2009	19,406 (44%)	8,996 (42%)	10,410 (46%)	
2010–2020	18,135 (41%)	9,123 (42%)	9,012 (40%)	
Tumor Grade				0.067
I/II	19,617 (44%)	9,651 (45%)	9,966 (44%)	
III/IV	24,606 (56%)	11,952 (55%)	12,654 (56%)	
Clinical stage				< 0.001
Localized	23,394 (53%)	13,239 (61%)	10,155 (45%)	
Regional	20,829 (47%)	8,364 (39%)	12,465 (55%)	
T stage				< 0.001
T1-T2	39,716 (90%)	20,056 (93%)	19,660 (87%)	
T3-T4	4,507 (10%)	1,547 (7%)	2,960 (13%)	
N stage				
N0	24,011 (54%)	13,527 (63%)	10,484 (46%)	< 0.001
N1-N3	20,212 (46%)	8,076 (37%)	12,136 (54%)	
Lymph node examined				< 0.001
0–15	33,884 (77%)	17,078 (79%)	16,806 (74%)	
16+	10,339 (23%)	4,525 (21%)	5,814 (26%)	
Estrogen receptor				0.084
Negative	14,044 (32%)	6,967 (32%)	7,076 (31%)	
Positive	30,179 (68%)	14,636 (68%)	15,544 (69%)	
Progesterone receptor				0.008
Negative	17,101 (39%)	8,552 (40%)	8,549 (38%)	
Positive	27,122 (61%)	13,051 (60%)	14,071 (62%)	
Chemotherapy				< 0.001
No	10,275 (23%)	6,926 (32%)	3,349 (15%)	
Yes	33,948 (77%)	14,677 (68%)	19,271 (85%)	
Development of SPMs				0.002
No	41,206 (94%)	20,373 (94%)	20,833 (92%)	
Yes	3,017 (7%)	1,230 (6%)	1,787 (8%)	

SPM, second primary malignancy; PORT, postoperative radiotherapy.

### PORT was associated with increased risks of SPMs in YWBCs

**Fig 1:**
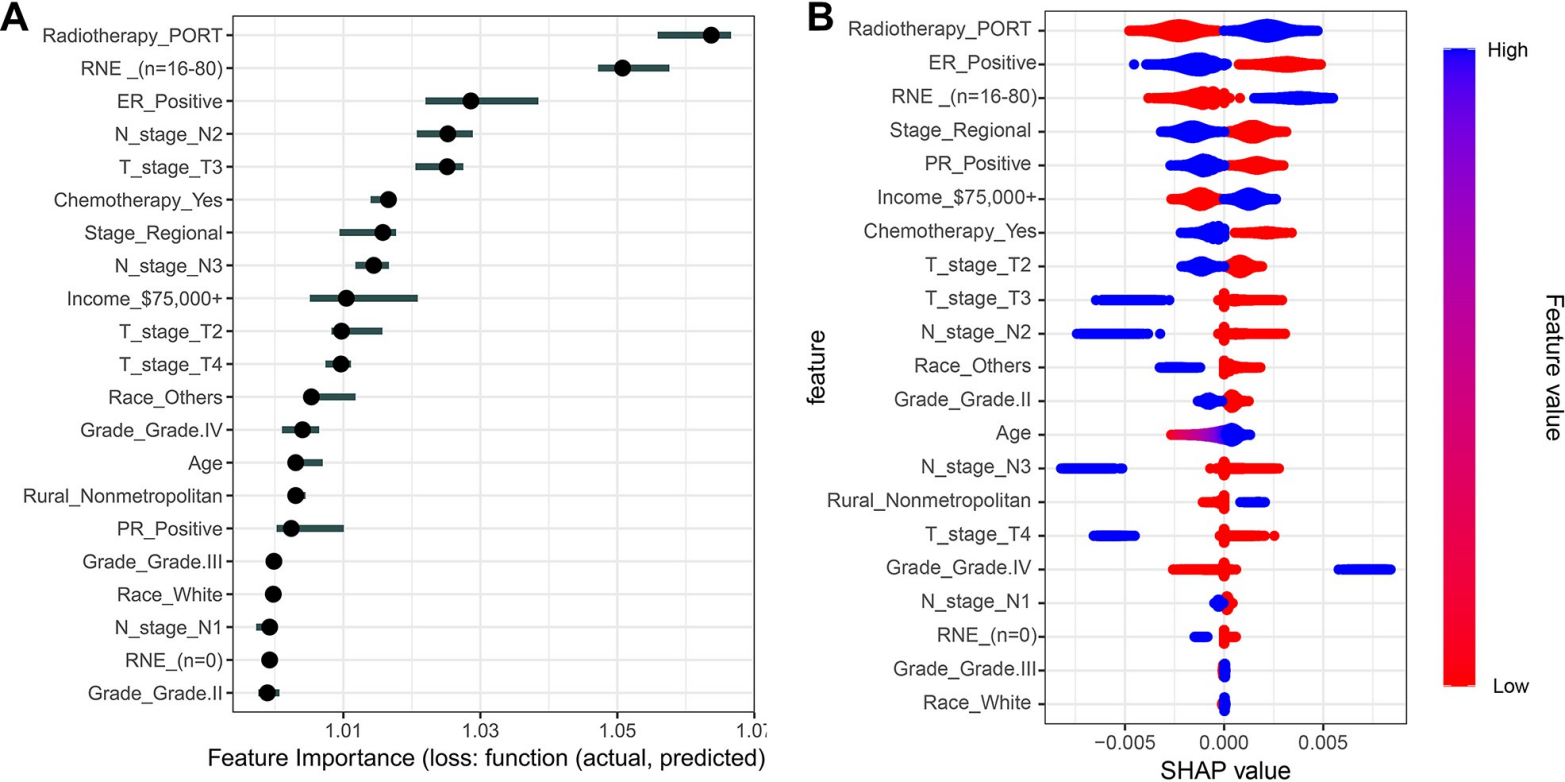
Ridge regression analysis revealed the effects of the clinical characteristics of patients with breast cancer on second primary malignancies. The feature importance (A) and SHAP values (B) of clinical characteristics for second primary malignancies. PORT, postoperative radiotherapy; RNE, regional nodes examined; ER, estrogen receptor; SHAP, Shapley additive explanations.

To fully clarify the correlation between YWBC counts and SPM risk, we used 8 machine learning components to evaluate the effects of the clinical characteristics of YWBC patients on SPMs. Among all the clinical characteristics, PORT contributed the most to the SPMs on the basis of the results of ridge regression (Fig 1A and 1B), XGBoost (S1A Fig), k-nearest neighbor (S1B Fig), light gradient boosting machine (S1C Fig), logistic regression (S1D Fig), and neural network (S1E Fig) analyses. S1F–S1G Fig shows the results of support vector machine and random forest, suggesting that age is the most powerful force driving SPMs. Thus, we further explored the correlation between PORT in YWBC patients and SPM risk.

**Fig 2:**
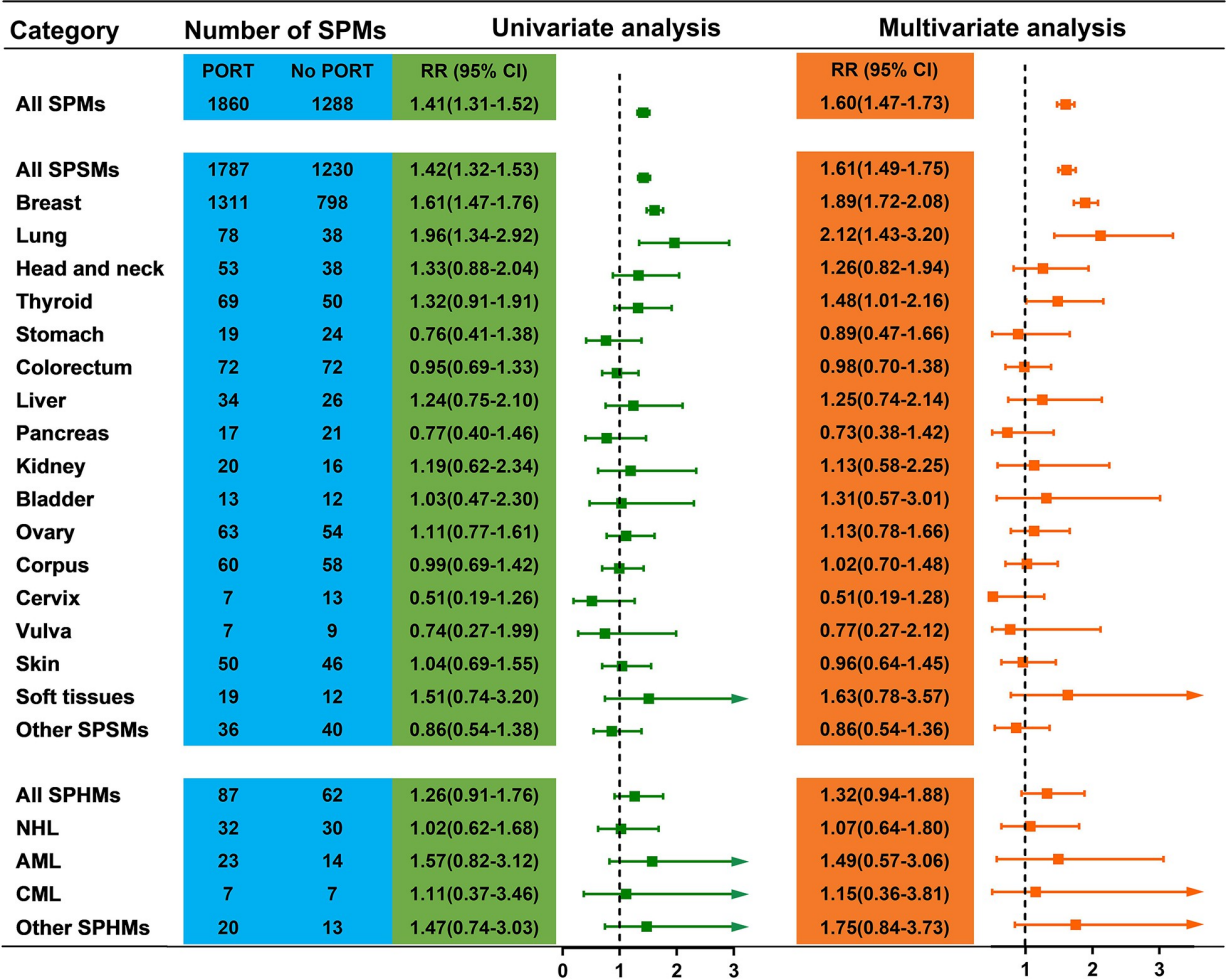
Relative risk for second primary malignancy in patients with breast cancer who did or did not receive PORT. RR, relative risk; PORT, postoperative radiotherapy; SPM, second primary malignancy; SPSM, second primary solid malignancy; SPHM, second primary hematologic malignancy; NHL, non-Hodgkin’s lymphoma; AML, acute myeloid leukemia; CML, chronic myeloid leukemia.

Fig 2 shows that for YWBC, the relative risk of all SPMs in the PORT group was greater than that in the no PORT group. (RR = 1.60; 95% CI: 1.47–1.73). We next quantified the relative hazards of SPHMs and SPSMs between the PORT group and the no PORT group, taking into account the varied latency times of SPHMs and SPSMs. YWBC cases receiving PORT were significantly correlated with higher risks of SPSMs (RR = 1.61; 95% CI: 1.49–1.75) but not SPHMs (RR = 1.32; 95% CI: 0.94–1.88). In particular, there were noteworthy associations between PORT-treated YWBC cases and increased risks of second primary breast cancer (RR = 1.89; 95% CI: 1.72–2.08), second primary lung cancer (RR = 2.12; 95% CI: 1.43–3.20), and secondary primary thyroid cancer (RR = 1.48; 95% CI: 1.01–2.16) (Fig 2). As shown in Fig 2, PORT and the risk of any subtype of SPHMs, such as myeloma and non-Hodgkin lymphoma, were not significantly correlated.

**Fig 3:**
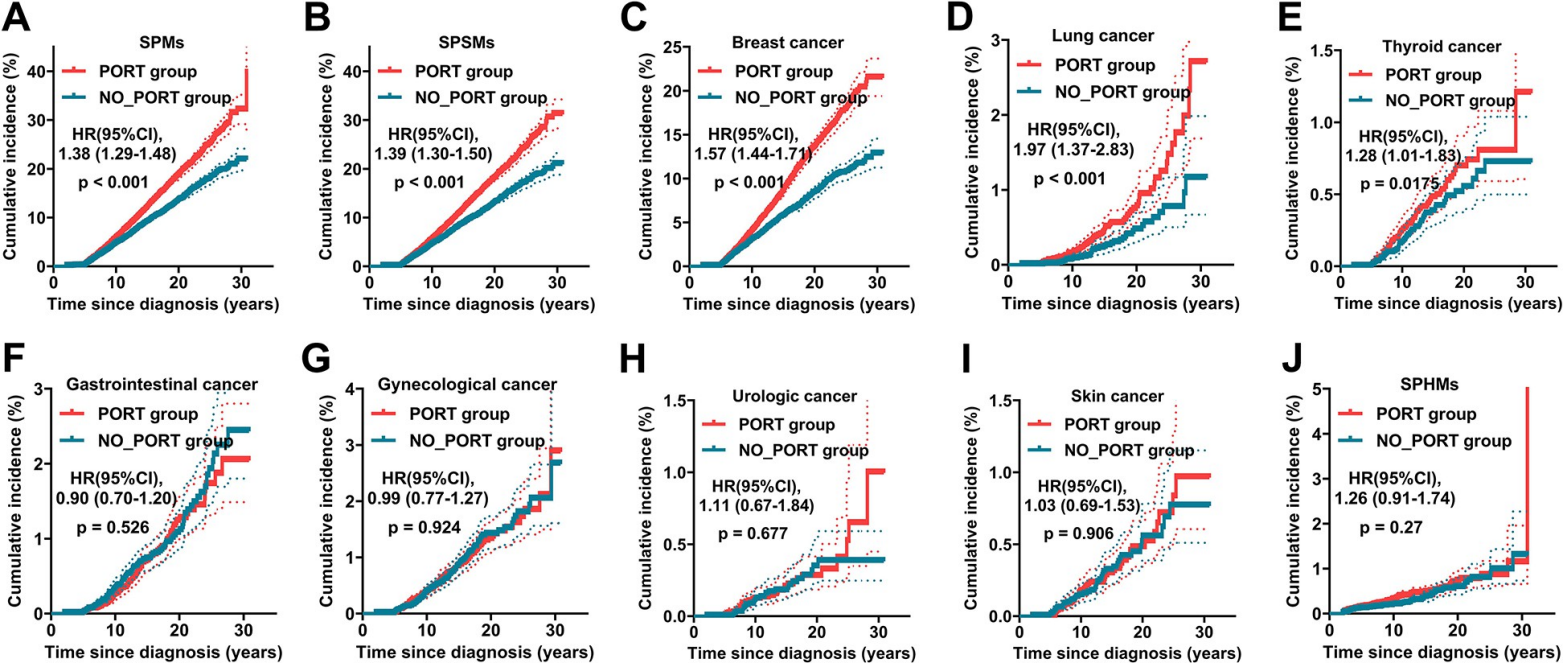
The cumulative incidence of second primary cancers in patients with breast cancer who received PORT or not. The cumulative incidence of all SPMs (A), SPSMs (B), breast cancer (C), lung cancer (D), thyroid cancer (E), gastrointestinal cancer (F), gynecological cancer (G), urologic cancer (H), skin cancer (I) and SPHMs (J) in breast cancer patients receiving PORT or not receiving PORT. PORT, postoperative radiotherapy; SPSM, second primary solid malignancy; SPHM, second primary hematologic malignancy.

The results of Fine‒Gray’s competing risk analysis are shown in Fig 3, which revealed a greater cumulative incidence of all SPMs (HR = 1.38; 95% CI: 1.29–1.48), SPSMs (HR = 1.39; 95% CI: 1.30–1.50), second primary breast cancer (HR = 1.57; 95% CI: 1.44–1.71), second primary lung cancer (HR = 1.97; 95% CI: 1.37–2.83) and second primary thyroid cancer (HR = 1.28; 95% CI: 1.01–1.83) in YWBC patients receiving PORT (Fig 3A–3E). There was no statistically significant difference in the cumulative incidence of second primary gastrointestinal cancer, second primary gynecological cancer, second primary urologic cancer, second primary skin cancer, or SPHMs between the PORT and no PORT groups (Fig 3F–3J).

**Fig 4:**
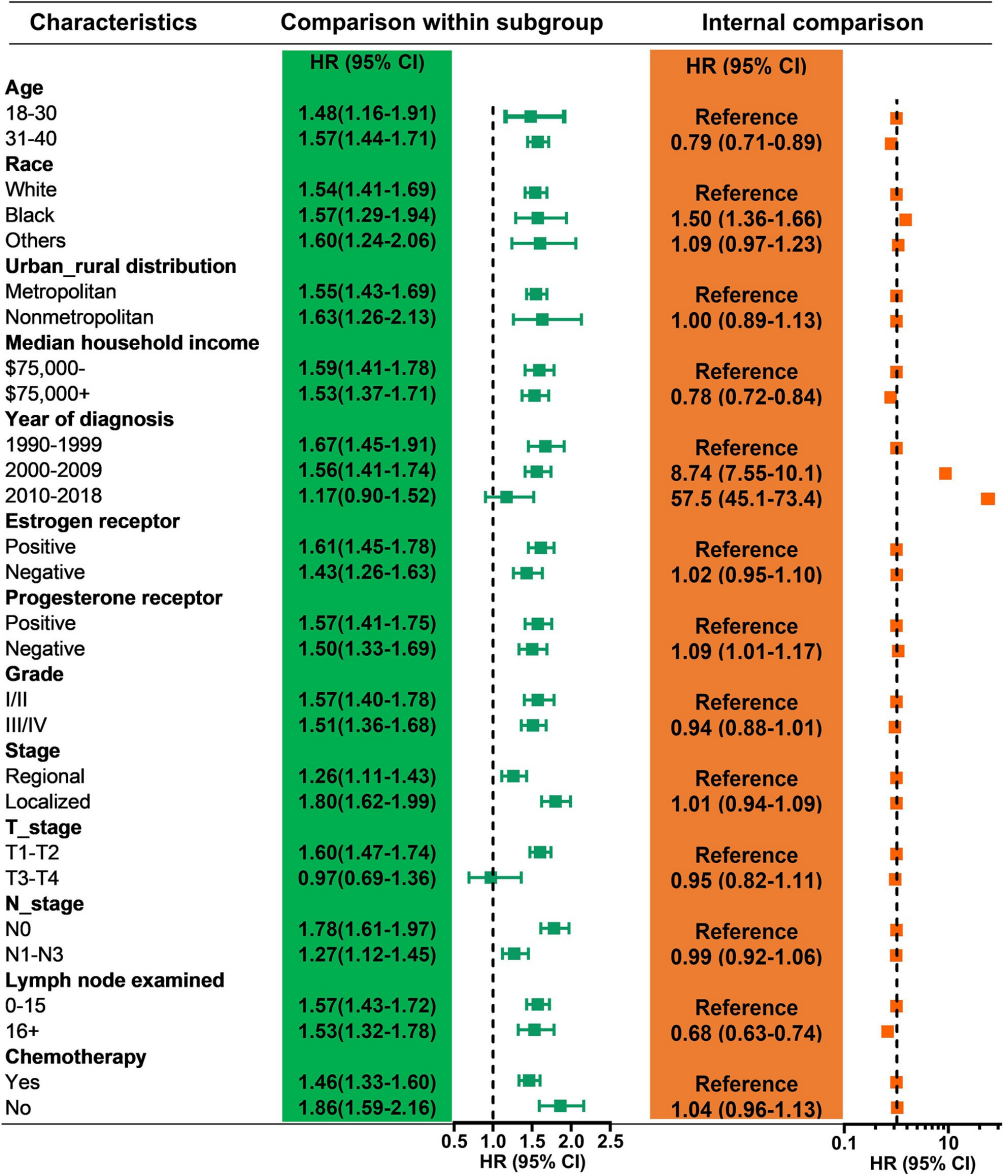
Relative risk for second primary malignancy in patients with different clinical characteristics of breast cancer. Comparison of the risk of SPM between subgroups according to the estimated HR. SPM, second primary malignancy; HR hazard ratio, SIR standardized incidence ratio, RR relative risk, PORT postoperative radiotherapy.

We also found that a greater risk of SPSMs in YWBC patients receiving PORT was suitable for each subgroup of YWBC patients, except for YWBC patients diagnosed from 2010–2020 and those with T3–T4 stage disease (Fig 4). Compared with YWBC patients aged 18–30 years, YWBC patients aged 31–40 years had a lower risk of developing SPMs (HR = 0.79; 95% CI: 0.71–0.89). Compared with white YWBC patients, black YWBC patients presented a greater risk of developing SPMs. A higher median household income (HR = 0.78; 95% CI: 0.72–0.84) and more lymph nodes examined (HR = 0.68; 95% CI: 0.63–0.74) indicated a lower risk of developing SPMs.

### Building and assessing the prediction nomogram

**Fig 5:**
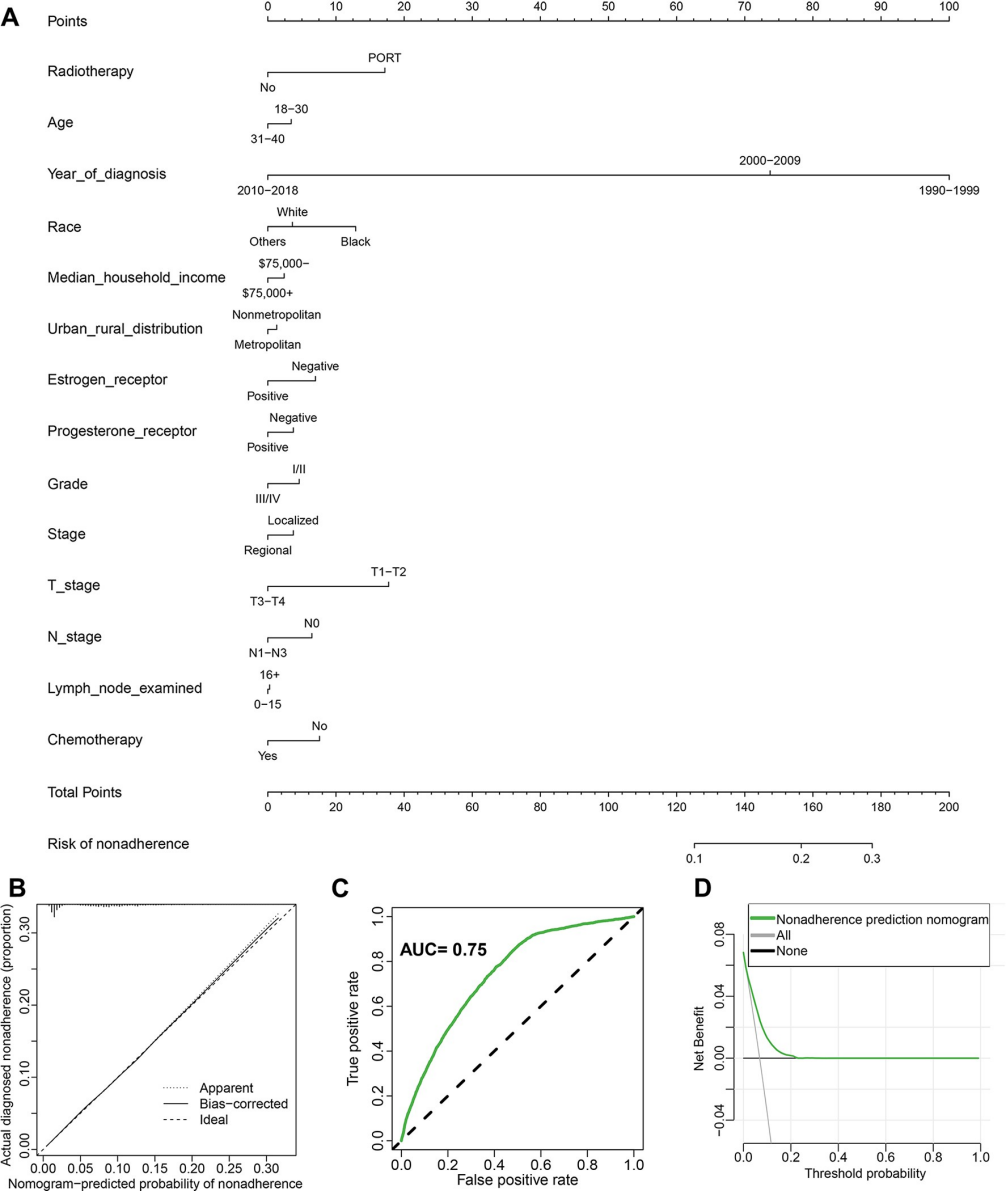
Predictive nomogram construction and assessment. (A) Nomogram constructed with clinicopathological characteristics. The nomogram’s performance was assessed via the calibration curve (B), area under the receiver operating characteristic curve (C), and decision curve analysis curve (D). AUC, area under the curve.

As shown in Fig 5A, using these clinicopathological criteria, we next built a prediction nomogram to predict the risk of SPMs. By adding the points for each variable, we can determine each patient’s total score and forecast the likelihood that SPMs will develop. The observed result and the expected probability in the calibration curves agreed fairly well, as Fig 5B illustrates. Fig 5C shows that the prediction nomogram’s area under the curve (AUC) was 0.75. Additionally, DCA indicated that the nomogram models had good clinical utility (Fig 5D).

## Discussion

To restrain tumor growth and prolong patient life, a variety of combination treatments, including radiotherapy and surgery, are already used in breast cancer patients. The negative effects of radiotherapy have decreased with technological developments, but radiotherapy may still increase the likelihood of developing SPMs, particularly in teenage cancer patients [23]. Using the largest population cohort available in the SEER cohort, the current study first thoroughly investigated the relationship between PORT and the likelihood of acquiring SPMs in YWBC at an early stage. The PORT of YWBC patients supported the increasing risk of developing SPSMs, such as thyroid, lung, and breast cancer. Longer follow-up periods were associated with an increased incidence of PORT-related SPSMs, such as thyroid, lung, and breast cancer. These findings suggest that these patients should be under long-term surveillance.

The current study focused on YWBC in localized and regional stages, who had more access to treatment and a longer life expectancy. Therefore, it is more reasonable to choose this part of the population for study. Adjuvant radiation was advised, according to ASCO guidelines, for patients with breast cancer with N1—3 disease but not for those with cT1–2cN0 disease [24]. In the present study, we focused on the relationship between YWBC in localized and regional stages who received PORT and the risk of SPMs, which may help map the impacts of going over instructions on PORT use in developing SPMs more thoroughly. Unlike other unfavorable occurrences, there is very little chance of obtaining SPMs. Therefore, a larger population and a longer follow-up period are needed for SPM studies. This new study’s SEER cohort, which included 44,223 breast cancer cases from 1975–2020, satisfied the sample and follow-up period requirements, yielding findings that may be trusted more.

Owing to the low incidence of SPM and complex influencing factors, a large population base and long follow-up time are needed for the study of SPM. Thus, there was a significant difference in the link between the risk of specific SPMs and PORT or radiotherapy of initial tumors. According to Bushra et al., the use of radiation therapy for breast cancer does not increase the likelihood of developing lung cancer [14]. However, another study demonstrated increased risks of contralateral breast cancer, lung cancer, and urinary system cancer in patients with breast cancer receiving adjuvant radiotherapy [16]. Patients with breast cancer who receive radiation therapy have an increased risk of developing a second esophageal carcinoma [25], which is different from the results of this study. A review including 16,705 patients suggested that adjuvant radiotherapy does not increase the risk of thyroid cancer [26]. Another study reported an increased risk of secondary thyroid cancer in patients with breast cancer receiving radiotherapy [15]. Thus, studies for PSM should strictly consider the inclusion and exclusion criteria, number of patients, definition of SPM, and methodology.

Moreover, our study also identified several risk factors that may contribute to SPM, including young age, black race, low median household income and fewer lymph nodes examined. A previous study revealed a correlation between black race and a greater risk of developing a second main contralateral breast cancer [27]. Another study also suggested a greater risk of SPM after colorectal cancer, with a lower median household income [28].

PORT is a crucial part of the treatment for breast cancer because it can increase patient survival. In our study, we found that PORT for breast cancer was correlated with a greater risk of second primary thyroid cancer, second primary lung cancer, and second primary breast cancer. Thus, standard adjuvant PORT should not be recommended for breast cancer patients with favorable histology and a low risk of relapse. For breast cancer patients who need PORT, clinicians should try to reduce radiation doses and protect other body tissues and organs from radiation. Moreover, as the risk of developing SPM is persistent in young-onset breast cancer patients receiving PORT, these patients should pay more attention to long-term and standardized follow-up.

It is important to acknowledge some limits. It is impossible to avoid selection bias and unmeasured confounding of SEER data. We were unable to acquire comprehensive PORT data, including PORT planning, dose, and type of PORT technique, which created a barrier to investigating the dose‒response relationship between PORT and the advancement of SPM. Our study’s findings were also impacted by a lack of data regarding medical insurance, family history of cancer, access to healthcare, genetic predispositions and environmental factors. Although several covariates were adjusted for in our study, there may still be unmeasured confounders that could affect the results, including lifestyle factors and other treatments received by the patients. Moreover, this analysis was performed on patients from the United States, which may not be suitable for other countries.

## Conclusion

Among all the clinical characteristics of YWBC patients, PORT contributed the most to SPMs. YWBC patients receiving PORT had a greater risk of SPSMs (thyroid, lung, and breast cancer), indicating the necessity of long-term surveillance of these patients. Standard adjuvant PORT should not be recommended for breast cancer patients with favorable histology and a low risk of relapse.

## Supporting information

S1 File Supplementary method for machine learning. (DOCX)

S1 Fig The machine learning components revealed the effects of the clinical characteristics of patients with breast cancer on second primary malignancies. The feature importance and SHAP values of the clinical characteristics of second primary malignancies based on the results of XGBoost (A), k-nearest neighbor (B), light gradient boosting machine (C), logistic regression (D), support vector machine (E), neural network (F), and random forest (G) methods. PORT, postoperative radiotherapy; RNE, regional nodes examined; ER, estrogen receptor; SHAP, Shapley additive explanations.
(JPG)

S1 Data (CSV)
