# Supplementary material for: Machine learning identifies the association between second primary malignancies and postoperative radiotherapy in young-onset breast cancer patients
Source: PLoS One. 2025 Feb 6;20(2):e0316722. doi: 10.1371/journal.pone.0316722 (PMC11801551; doi:10.1371/journal.pone.0316722)
Supplement: S1 File — (DOCX) [file pone.0316722.s001.docx]

**Supplementary method.**

The SEER dataset was then randomly divided into a training set (70%) and a test set (30%). Least absolute shrinkage and selection operator (LASSO) regression analysis was used to filter 13 clinical variables based on 10-fold cross-validation. Three methods, random forest (RF) regressor, extreme gradient boosting (XGBoost) classifier, and ridge regressor, were used to analyze variable importance ranking. The top 10 variables were selected by taking the intersection of the Wayne plots. Additionally, seven ML algorithms, including Ridge regression, XGBoost, k-nearest neighbor, light gradient boosting machine, logistic regression, support vector machine, neural network, random forest were used to predict identify the risk factors for SPM. On the training set, we used k-fold cross-validation and a resampling approach (k=10). We used the validation set to optimize the model parameters and the test set to evaluate the system performance. Three measures of model quality—discrimination, calibration and clinical utility—were used to assess the clinical value of the prediction model, and exact recall curve analysis was used to evaluate model discrimination. The degree of calibration and the difference between model predictions and actual events were measured using calibration plots. The metrics of the confusion matrix were assessed to calculate the mean precision, accuracy, sensitivity, specificity, and F-value scores of the models. There are limitations in the interpretation of the results of ML techniques. Lundberg’s Shapley additive explanation (SHAP) method is based on game theory and is used to interpret results of any ML model. It is a reliable, rapid, and cost-effective approach. It is crucial to note that the SHAP method assesses the relevance of every predictor variable based on its SHAP value. A high SHAP value has a positive impact on the output of the ML model, while a low SHAP value has the opposite effect. Ultimately, a thorough analysis was completed for the assimilation of seven variables. This resulted in the creation of an online forecasting model which can be readily used by healthcare practitioners.
