# Supplementary figures and images for: Machine learning identifies the association between second primary malignancies and postoperative radiotherapy in young-onset breast cancer patients
Source: PLoS One. 2025 Feb 6;20(2):e0316722. doi: 10.1371/journal.pone.0316722 (PMC11801551; doi:10.1371/journal.pone.0316722)

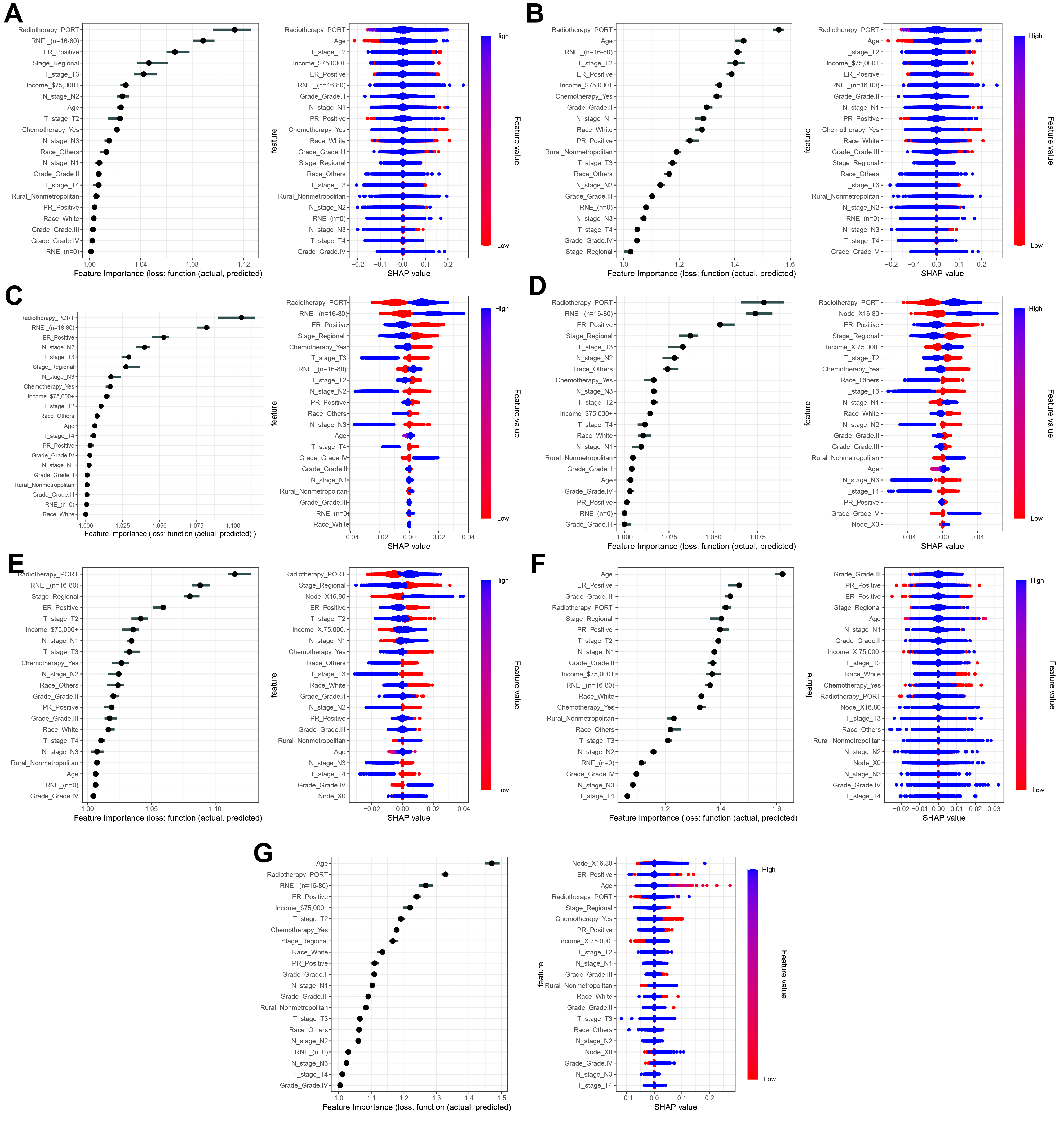

Supplement: S1 Fig — The feature importance and SHAP values of the clinical characteristics of second primary malignancies based on the results of XGBoost (A), k-nearest neighbor (B), light gradient boosting machine (C), logistic regression (D), support vector machine (E), neural network (F), and random forest (G) methods. PORT, postoperative radiotherapy; RNE, regional nodes examined; ER, estrogen receptor; SHAP, Shapley additive explanations. (JPG) [file pone.0316722.s002.jpg]
